# Supplementary material for: MetaQTL: a package of new computational methods for the meta-analysis of QTL mapping experiments
Source: BMC Bioinformatics. 2007 Feb 8;8:49. doi: 10.1186/1471-2105-8-49 (PMC1808479; doi:10.1186/1471-2105-8-49)
Supplement: Additional File 7 — MetaQTL Package : jar file and tutorial. This Zip archive contains both the MetaQTL JAR file and the files of the tutorial. [file 1471-2105-8-49-S7.zip › org.inra.metaqtl/doc/org/metaqtl/bio/package-summary.html]

org.metaqtl.bio


|  |  |  |
| --- | --- | --- |
| **Overview** \| **Package** \| Class \| **Use** \| **Tree** \| **Deprecated** \| **Index** \| **Help** | |  |
| **PREV PACKAGE**   **NEXT PACKAGE** | **FRAMES**    **NO FRAMES**     **All Classes** |  |


---

## Package org.metaqtl.bio

| **Interface Summary** | |
| --- | --- |
| **IBioAdaptable** |  |
| **IBioAdapter** |  |
| **IBioAllele** | Class Description Here |
| **IBioCross** | Class Description Here |
| **IBioCrossTypes** | Class Description Here |
| **IBioEntity** | Class Description Here |
| **IBioEntityTypes** | Class Description Here |
| **IBioGenome** |  |
| **IBioIndividual** | Class Description Here |
| **IBioIndividualTree** | Class Description Here |
| **IBioLGroup** |  |
| **IBioLocus** |  |
| **IBioOntology** | This interface defines some rules to handle a ontology. |
| **IBioOntologyTerm** | This class defines a term of an ontology. |
| **IBioPopulation** | Class Description Here |
| **IOntologyTermProperties** |  |

---


|  |  |  |
| --- | --- | --- |
| **Overview** \| **Package** \| Class \| **Use** \| **Tree** \| **Deprecated** \| **Index** \| **Help** | |  |
| **PREV PACKAGE**   **NEXT PACKAGE** | **FRAMES**    **NO FRAMES**     **All Classes** |  |


---
